# The Effects of TiO_2_ Nanodot Films with RGD Immobilization on Light-Induced Cell Sheet Technology

**DOI:** 10.1155/2015/582359

**Published:** 2015-08-31

**Authors:** Meng-Liu Yu, Meng-Fei Yu, Li-Qin Zhu, Tian-Tian Wang, Yi Zhou, Hui-Ming Wang

**Affiliations:** ^1^The Affiliated Hospital of Stomatology, College of Medicine, Zhejiang University, Hangzhou, Zhejiang 310006, China; ^2^The First Affiliated Hospital, College of Medicine, Zhejiang University, Hangzhou, Zhejiang 310006, China; ^3^Department of Materials Science and Engineering, State Key Laboratory of Silicon Materials, Zhejiang University, Hangzhou, Zhejiang 310027, China

## Abstract

Cell sheet technology is a new strategy in tissue engineering which could be possible to implant into the body without a scaffold. In order to get an integrated cell sheet, a light-induced method via UV365 is used for cell sheet detachment from culture dishes. In this study, we investigated the possibility of cell detachment and growth efficiency on TiO_2_ nanodot films with RGD immobilization on light-induced cell sheet technology. Mouse calvaria-derived, preosteoblastic (MC3T3-E1) cells were cultured on TiO_2_ nanodot films with (TR) or without (TN) RGD immobilization. After cells were cultured with or without 5.5 mW/cm^2^ UV365 illumination, cell morphology, cell viability, osteogenesis related RNA and protein expression, and cell detachment ability were compared, respectively. Light-induced cell detachment was possible when cells were cultured on TR samples. Also, cells cultured on TR samples showed better cell viability, alongside higher protein and RNA expression than on TN samples. This study provides a new biomaterial for light-induced cell/cell sheet harvesting.

## 1. Introduction

Tissue engineering, in which cells play a fundamental role, is responsible for tissue repair [1]. The most common method for harvesting cells is by enzymatic treatment, wherein extracellular matrix (ECM) proteins are digested by trypsin, resulting in the release of cultured cells from culture dishes. However, this approach can damage important transmembrane proteins such as cytoskeletal elements, signaling molecules, and receptors [2]. In addition, the cleavage of these proteins has been proven to lead to loss of functional expression [3].

As has been previously demonstrated, ECM proteins adhere easily onto hydrophobic surfaces, as opposed to hydrophilic surfaces [4] in which the temperature-induced cell harvest method has been developed. This method could be used to harvest single cells or a layer of confluent cells, and it has a less damaging effect than enzymatic treatment because it preserves ECM and transmembrane proteins [5]. The layer of confluent cells has since been developed as a new strategy in tissue engineering, referred to as cell sheet technology [6]. It is difficult to obtain hard tissue cell sheets by the temperature-induced method, as temperature change can accelerate cell senescence of hard tissue cells [7]. Besides the temperature-induced method, there are other applicable harvesting methods, although they all have their respective drawbacks. In the pH change-induced method [8], the regulation of pH value could result in local deviation and decreased cell proliferation and viability [9]. In the electricity-induced method [10] and magnetism-induced method [11], materials of the layer are found on detached cells or cell sheets and influence the result of subsequent experiments. Recently, the light-induced method, which is based on the changes of surface wettability of certain materials [12], has been developed and provides a more convenient approach for cell harvesting [13]. The light resource (365 nm ultraviolet) used in the method has been proven as a safe resource for biologic organisms [14, 15] and has been demonstrated to obtain numerous types of cell sheets successfully [13].

Another key factor of cell sheet engineering is the material that enables cell adhesion and detachment. Considering the importance of cell-cell interaction for cell attachment, TiO_2_ nanodot films could be functionalized to stimulate related protein expression. The Arg-Gly-Asp (RGD) peptide is relevant to cell adhesion, proliferation, and many ECM proteins [16]. A peptide containing the RGD motif could accelerate cell attachment, extension of primary bone-derived cells [17], cellular protein expression [18–20], and bone formation on titanium [21].

This study aimed to examine the RGD immobilized surface used in the light-induced method for cell sheet technology. Accordingly, we verified light-induced cell sheet detachment on the RGD immobilized surface. We also investigated the effects of RGD on cell adhesion, proliferation, cell viability, and osteogenesis protein expression.

## 2. Materials and Methods

### 2.1. Preparation and Characterization of TiO_2_ Nanodot Films and Immobilization of RGD

TiO_2_ nanodot films were prepared by the phase-separation-induced self-assembly method on quartz substrates [22]. Briefly, acetylacetone (Lingfeng Chemical Reagent, AR, >99%), deionized water, and titanium tetrabutoxide (TBOT, Sinopharm Chemical Reagent, CP, >98%) were dissolved in 100% ethanol at the ratio of 0.3 : 1 : 1. Polyvinyl pyrrolidone (PVP, K30, Sinopharm Chemical Reagent, AR, >99%) was then added with 4% mass ratio to obtain a homogeneous sol-precursor. After spin-coating on Ti substrates at 7000 rpm for 40 s and heating at 500°C for one hour, TiO_2_ nanodot film was obtained.

RGD peptides were dissolved in PBS to obtain 0.5 mg/mL solution. Then autoclaved TiO_2_ nanodot films were immersed in the solution for 24 h at 37°C in a sterile environment. Samples were aired and stored in 4°C before use.

### 2.2. Cell Culture

Mouse preosteoblastic MC3T3-E1 cells (CRL-2594, ATCC) were used in this study. Alpha-modified minimum essential media (*α*MEM, Gibco) supplemented with 10% fetal bovine serum (FBS, PAA, Australia), 1% sodium pyruvate (Gibco), 1% antibiotic solution containing 10,000 units/mL penicillin and 10,000 *μ*g/mL streptomycin (Gibco), and 1% MEM nonessential amino acids (Gibco) were used for the cell culture.

### 2.3. Cell Attachment and Detachment Assay

#### 2.3.1. Cell Morphology Assay

MC3T3-E1 cells were seeded on 1 × 1 cm^2^ TiO_2_ nanodots films (TN) or TiO_2_ nanodots films with RGD peptides (TR) in 24-well plates at a density of 1 × 10^5^ cells/cm^2^. Cell morphology was observed by a phase-contrast microscope (CKX41, Olympus, Japan) 1 hour, 3 hours, 1 day, and 3 days after seeding.

#### 2.3.2. Cell Attachment Ratio Assay

After cells were seeded on TN or TR for 3 hours, 1 day, and 3 days, the cell counting kit-8 (cck-8, Dojindo, Japan) assay was used to measure the cells attachment on the samples' surface.

#### 2.3.3. UV Resources and Illumination Method for Detachment

A cold LED UV light which could eliminate heat interference with 365 nm wavelength was used in this study for cell sheets detachment. After cells were cultured for 1 day, the samples were rinsed gently with PBS three times. The power of 5.0 mW/cm^2^, 5.5 mW/cm^2^, and 6.0 mW/cm^2^ UV light was used with an illumination time of 30 min. PBS was used for rinsing the samples' surface. The cell counting kit-8 (cck-8, Dojindo, Japan) assay was used to measure the residual cells on the samples' surface. The surfaces of blank polystyrene (PS) samples were also measured as the control group.

#### 2.3.4. SEM for Cell Morphology

After being cultured for 1 day, the cells were illuminated under 5.5 mW/cm^2^ UV365 for 30 min. Samples without UV365 illumination were used as the control group. After being fixed with 2.5% glutaraldehyde at 4°C overnight and undergoing dehydration in a series of ethanol solutions, they were immersed in HMDS for 10 min and air-dried before observation by a SEM (SU-70, Hitachi, Japan) [23].

### 2.4. Cell Sheet Viability

#### 2.4.1. Cell Sheet Detachment and Reattachment Assay

MC3T3-E1 cells were seeded as mentioned above. After being cultured for 7 days to form a monolayer, cells were illuminated under 5.5 mW/cm^2^ power of UV365 for 30 min to obtain cell sheets from the TN and TR surface [13]. The obtained cell sheets were plated on 24-well cell culture dishes to evaluate their reattachment ability at day 1 and day 3.

#### 2.4.2. Viability Assay

After being cultured for 7 days, cells were illuminated under 5.5 mW/cm^2^ power of UV365 for 30 min. Annexin V-fluorescein isothiocyanate (FITC, 0.1 *μ*g/mL) and propidium iodide (PI, 0.5 *μ*g/mL) (Invitrogen) were used to analyze cell apoptosis and death using an FC500 flow cytometer (Beckman Coulter, USA).

#### 2.4.3. Live-Dead Staining of Harvested Cell Sheets

The Hoechst-PI double-labeled method was used to assess cell viability. Samples were cultured for 7 days. After illumination under 5.5 mW/cm^2^ power of UV365 for 30 min, samples were rinsed with PBS three times and incubated with Hoechst 33342 in the concentration ratio of 1 : 2000 with PBS for 5 min at room temperature in the dark. The samples were then incubated with PI at the concentration ratio of 1 : 200 with PBS at room temperature in the dark after being rinsed three times with PBS. After being rinsed with PBS another three times, cell sheets were observed with a fluorescence microscope (IX81, Olympus, Japan) and figures were analyzed with Image-Pro 6.0 software (Media Cybernetics, USA). A piece of cell sheet with the same staining procedure after 5 minutes of treatment in 65°C water bath was observed as the negative control.

### 2.5. Immunostaining

Samples were cultured for 7 days, fixed in 4% paraformaldehyde at room temperature for 20 min, and rinsed with PBS before permeabilization by 0.3% Triton X-100 (Sigma, USA) in PBS for 15 min on ice. After being rinsed with PBS, samples were blocked by 2.5% BSA (Sigma, USA) in PBS at room temperature for 60 min. The cell sheets were incubated with Cadherin primary antibody (ab6528, 1 : 100, Abcam, UK) in 1% BSA at 4°C overnight after rinsing three times with PBS. Cell sheets were incubated with a secondary antibody (Alexa Fluor 488 Goat Anti-Mouse IgG (H+L) antibody, 1 : 400, Invitrogen, USA) at room temperature and kept in the dark for 60 min. Cell nuclei were assessed by Hoechst 33342 as mentioned above. Samples were observed by laser scanning confocal microscopy (IX81, Olympus, Japan). Images and figures were analyzed by Image-Pro 6.0 software (Media Cybernetics, USA).

### 2.6. Quantitative Real-Time PCR

MC3T3-E1 cells were cultured on TN or TR samples for 7 days. After being illuminated under 5.5 mW/cm^2^ power of UV365 for 30 min, the total RNA of each sample was extracted using Trizol Reagent (Invitrogen, USA). The concentration and purity of total RNA were measured by a microultraviolet spectrophotometer (SMA1000, Merinton, USA). Then, RNA was reverse-transcribed to cDNA using PrimeScript II 1st Strand cDNA Synthesis Kit (Takara, Japan), and iTaq Universal SYBR Green Supermix (Bio-Rad, USA) was used for PCR reactions. The primer sequences are shown in Table 1. The thermocycling conditions were followed according to the manufacturer's instructions in the Chromo-4 Real-Time PCR System (Bio-Rad, USA). Calculation of the gene copy number was carried out using the −ΔΔCt method [24].

### 2.7. Western Blot Analysis

MC3T3-E1 cells were cultured on TN or TR samples for 7 days. After being illuminated under 5.5 mW/cm^2^ power of UV365 for 30 min, cells of each sample were lysed using cell lysis buffer (Cell Signaling, Beverly, MA) and the total protein was collected. Proteins were separated on 10% sodium dodecyl sulfate-polyacrylamide gel electrophoresis (SDS-PAGE) and were transferred to polyvinylidene fluoride (PVDF) membranes (Bio-Rad, Hercules, CA). Membranes were incubated with primary antibodies of GAPDH (Cell Signaling, Beverly, MA), alkaline phosphatase (ALP, ab65834, Abcam, UK), Collagen I (ab21286, Abcam, UK), and runt-related transcription factor 2 (Runx2, ab23981, Abcam, UK) at 4°C overnight, respectively. Membranes were incubated with HRP-anti-Rb antibody (Lot number: 050884, KPL) for 1 h at room temperature, and bands were shown by the enhanced chemiluminescence (ECL) solution (Thermo Scientific, USA). Band densities were analyzed by Quantity One software (Bio-Rad, Hercules, USA).

### 2.8. Statistical Analysis

All data were expressed as means ± standard deviation. They were analyzed using the SPSS 17.0 software package by factorial ANOVA and Scheffe's post hoc test. Differences were considered statistically significant at *P* < 0.05. All experiments were performed at least three times.

## 3. Results

### 3.1. Cell Attachment

#### 3.1.1. Cell Attachment Ability

**Figure 1:**
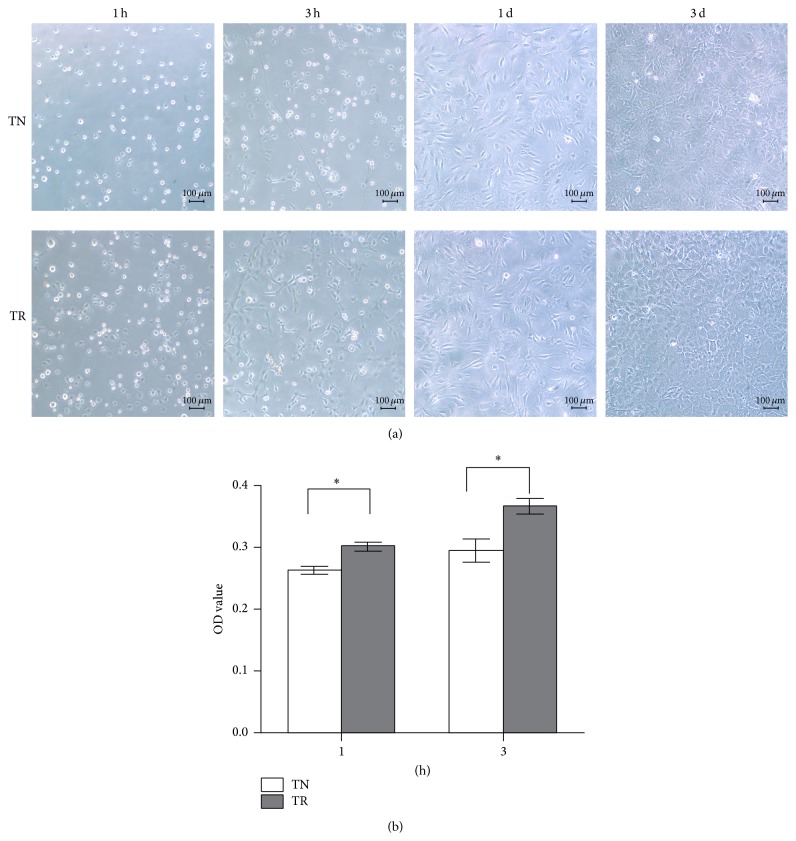
(a) Morphology of MC3T3-E1 cells cultured on TN samples or TR samples after seeding for intended time observed by inverted microscope. Scale bar: 100 *μ*m. (b) Cell number counting after MC3T3-E1 seeded 1 hour or 3 hours by cck-8 kit. ^*∗*^ *P* < 0.05.

The ability of cell attachment and cell morphology on TN or TR samples were estimated to determine whether the materials would be harmful to cells. The result showed that cells on TR samples adhered faster than on TN samples 1 h or 3 h after seeding. There was no significant difference between these two materials 1 day after seeding (Figure 1(a)). For the cck-8 measurement, the result was coincident with the morphology result (Figure 1(b)). The data of cell seeded after 1 day or 3 days was not shown in the figure.

#### 3.1.2. Immunostaining

**Figure 2:**
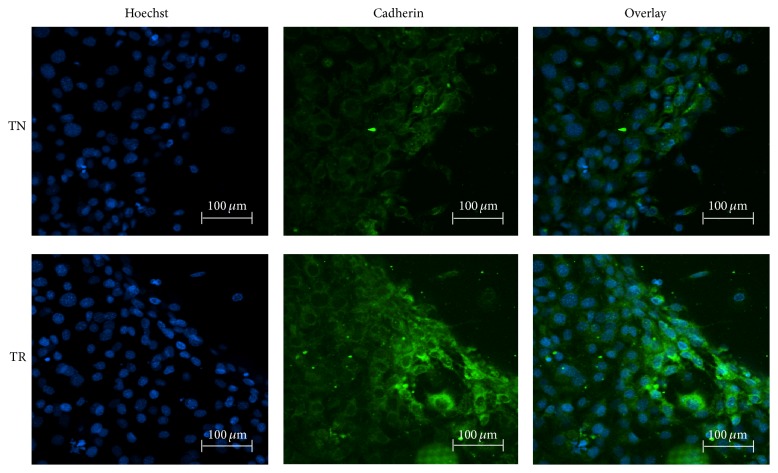
Immunostaining of Cadherin from MC3T3-E1 cell sheets cultured on TN samples or TR samples for 7 days. Scale bar: 100 *μ*m.

The display of Cadherin of cell sheets cultured on TN or TR samples is shown in Figure 2. We found that both cell sheets showed Cadherin existence, while cell sheets cultured on TR samples exhibited a stronger fluorescence signal than those on TN samples extracellularly.

### 3.2. Cell Sheet Osteogenesis Related Ability

#### 3.2.1. Quantitative Real-Time PCR

**Figure 3:**
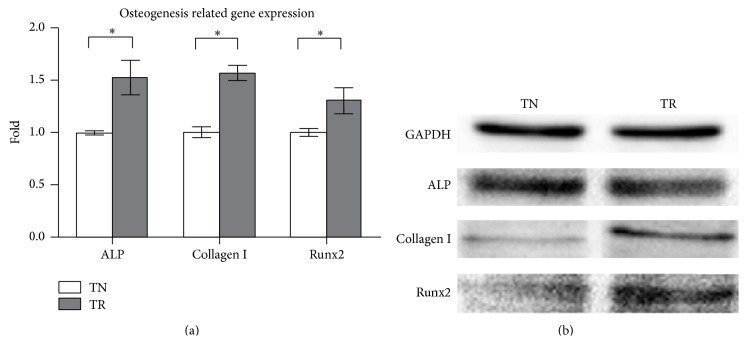
(a) Q-PCR analysis of ALP, Collagen I, and Runx2 RNA expression level of MC3T3-E1 cells cultured after 7 days. GAPDH was used as internal reference. ^*∗*^ *P* < 0.05. (b) Western blot analysis of GAPDH, ALP, Collagen I, and Runx2 expression of MC3T3-E1 cells after cultured for 7 days.

The 2^−ΔΔCT^ value was used to analyze the RNA expression. ALP, Collagen I, and Runx2 genes showed a higher transcription level when cells were cultured on TR samples for 7 days, as compared to those cultured on TN samples (Figure 3(a)) (*P* < 0.05).

#### 3.2.2. Western Blot

After being cultured for 7 days, Western blot analysis revealed a higher expression of Collagen I and Runx2 on TR samples than on TN samples. The expression level of ALP in two different samples showed no significant difference (Figure 3(b)).

### 3.3. Cell and Cell Sheet Detachment and Reattachment

#### 3.3.1. Efficiency of UV365 on Cell Detachment

**Figure 4:**
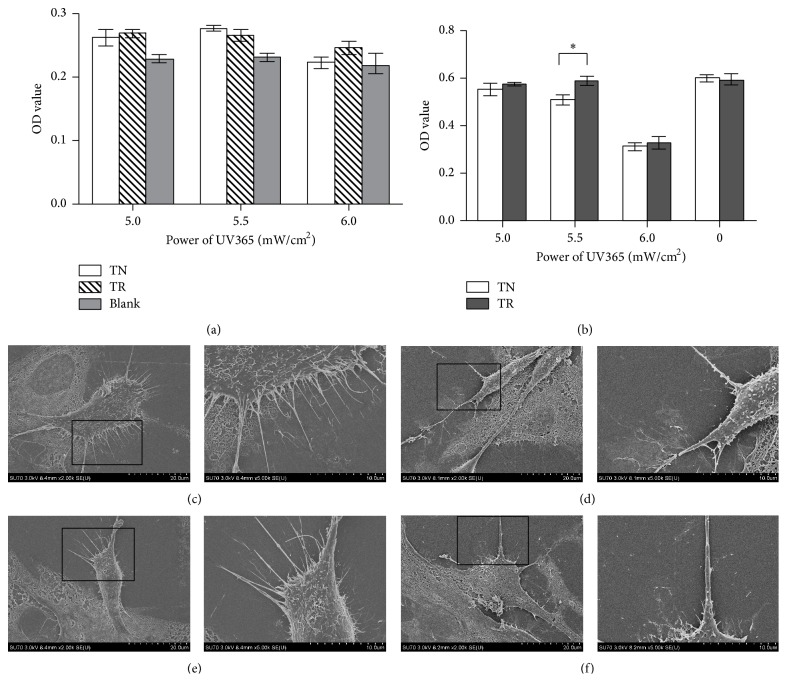
(a) Residual MC3T3-E1 cells on samples after UV365 illumination and cell detachment technology tested by cck-8 kit. (b) Lived MC3T3-E1 cells on samples after UV365 illumination without cell detachment operation tested by cck-8 kit. ^*∗*^ *P* < 0.05. (c) Morphology of MC3T3-E1 cells cultured on TR samples before UV365 illumination. (d) Morphology of MC3T3-E1 cells cultured on TR samples after UV365 illumination. (e) Morphology of MC3T3-E1 cells cultured on TN samples before UV365 illumination. (f) Morphology of MC3T3-E1 cells cultured on TN samples after UV365 illumination. Scale bar: 10 *μ*m or 20 *μ*m.

After 1-day culture on TN and TR samples, with illumination with the intended UV light, the residual cells on each sample were measured. The result indicated that the power of 5.0 mW/cm^2^, 5.5 mW/cm^2^, and 6.0 mW/cm^2^ UV light in this study could cause cells to detach from materials after the detachment operation (Figure 4(a)). Comparing the OD value of either TN or TR samples with the blank controls, the residual cell number was negligible (*P* > 0.05). The result of cell viability after UV365 illumination for 30 min (Figure 4(b)) revealed that there was a significant difference after illumination under the power of 5.5 mW/cm^2^ of UV365 (*P* < 0.05). The OD value of TR samples was higher than the ones on TN samples, implying that there were more living cells on TR samples after illumination. Moreover, there was no obvious difference when the illumination power was increased to 6.0 mW/cm^2^ (*P* > 0.05).

#### 3.3.2. SEM Observation

The images of cells without UV365 illumination seeded either on TN samples or on TR samples (Figures 4(c) and 4(e)) showed that the pseudopodia did exist around the cells and were fully extended. After illumination of the intended power of UV365, the image showed that the number of pseudopodia around the cells seeded on TN and TR samples (Figures 4(d) and 4(f)) was decreased, and residual pseudopodia were retracted when compared to cells not receiving UV365 illumination. In addition, the cell volume was smaller than that observed without UV365 illumination.

#### 3.3.3. Viability by Flow Cytometry Measurements and Live-Dead Staining

**Figure 5:**
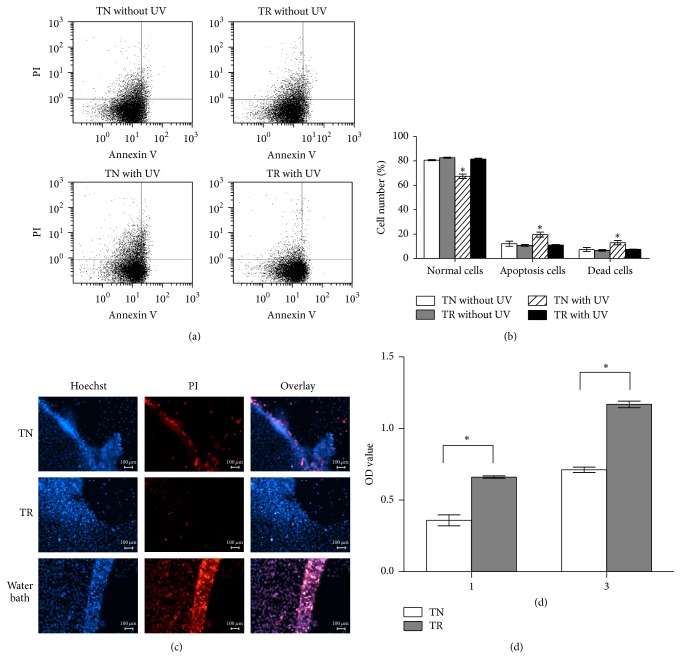
(a) Cell apoptosis and death of samples were tested by flow cytometry after culturing for 7 days. (b) Cell number of normal, apoptosis, or death cells has been counted. There is statistic difference when TN with UV group compared to any of the other three groups. ^*∗*^ *P* < 0.05. (c) Viability of MC3T3-E1 cell sheets cultured on TN samples or TR samples staining by Hoechst-PI double-labeled method after UV365 for 30 min. MC3T3-E1 cell sheets cultured on PS samples after 65°C water bath were stained as control group. Scale bar: 100 *μ*m. (d) Attachment and proliferation ability of reseeded MC3T3-E1 cells were tested by cck-8. The abscissa indicated the cultured days for cells. ^*∗*^ *P* < 0.05.

We performed flow cytometry and live-dead staining to investigate the survival of MC3T3-E1 cells detached under the power of 5.5 mW/cm^2^ of UV365 illumination. The original flow cytometry images and the result of its cell number counting are shown in Figures 5(a) and 5(b). Compared to cells from TN samples, there was a much lower incidence of apoptosis and dead cells in TR samples after 5.5 mW/cm^2^ of UV365 illumination (*P* < 0.05). There was no significant difference in cell viability from TR samples after UV 365 illumination as compared to cell viability without UV 365 illumination (*P* > 0.05).

To follow up on this finding, we conducted the Hoechst-PI double-labeled method to observe the survival of detached cell sheets. As shown in Figure 5(c), the number of dead cells obtained from TN samples was increased compared to the number of cells from TR samples.

#### 3.3.4. Cell Sheet Reattachment Assay

The reattachment behavior of MC3T3-E1 cells is shown in Figure 5(d). After illumination under the power of 5.5 mW/cm^2^ of UV365, cell sheets obtained from both TN samples and TR samples had the ability to reattach on the normal 24-well plates. The number of reattached cells of cell sheets from TR samples was more than that from TN samples after being cultured for 1 day and 3 days (*P* < 0.05).

## 4. Discussion

Previous studies have shown that biologically active molecules can attach to titanium surfaces through adsorption, covalent binding, self-organizing organic layers, and nanomechanical incorporation. The immobilized method of RGD peptides in this study results from adsorption ranging from physisorption to chemisorption [25]. In addition, the ability to promote cell adhesion of RGD peptides has been proven in this approach [26]. It was confirmed in this study that RGD peptides could improve the initial attachment of MC3T3-E1 cells to the samples' surface. No significant difference was observed on either TN samples or TR samples after 1-day incubation. An explanation may be that this time period is sufficient enough to overcome the function of RGD peptides on promoting initial cell adhesion and proliferation.

It has been known that the Cadherin junction is a strong mediator of cell survival [20]. The action of Cadherin involves cell-to-cell adhesion and interference with intracellular signaling [27]. In addition, Cadherin-induced pathways could strengthen the capacity of cells to resist apoptosis and death [28]. The RGD peptide plays a role in promoting the expression of Cadherin [20]. Therefore, the fluorescence signal of Cadherin was higher on TR samples than on TN samples, which likely is in response to the presence of RGD. Additionally, expression of Cadherin could reduce cell apoptosis, which could be a reason why less apoptosis occurred on TR samples with flow cytometry analysis [29].

ALP is an early osteogenic marker, which could correspond to cell differentiation [30]. MC3T3-E1 cells have the ability to produce Collagen I and differentiate into osteoblast-like cells. Collagen I is the fundamental component of bone matrix which can induce calcification [31]. Runx2 is known as a major factor for osteogenesis and functions as a primary effector in regulating bone differentiation markers [32]. After cells were cultured for 7 days, the result of Q-PCR showed a higher expression of RNA of ALP, Collagen I, and Runx2. The Western blot analysis showed higher protein expression of Collagen I and Runx2 on TR samples than on TN samples which was coincident with Q-PCR's result. However, Western blotting analysis revealed that there was no significant difference in ALP expression. This could be explained by the fact that transcription occurs earlier than translation, which itself is controlled by the expression of mRNA [33]. Therefore, the increase of RNA expression levels would appear earlier than the increase of protein expression levels. It has been proven that the RGD peptide could improve the mRNA and protein expression related to cell differentiation and calcification [34, 35] which has been confirmed again in this study. Also, RGD is a favorable peptide for surface modification on two-dimensional or three-dimensional biomaterials in osteogenesis [36]. Therefore, this biomaterial could be used as a further osteogenic biomaterial.

Titanium dioxide is one of the best candidates that have the light-induced superhydrophilic property [12]. And UV365 was a reasonable light source for cell detachment from TiO_2_ nanodot films because the illumination of UV365 could change the TiO_2_ surface status from hydrophobicity to hydrophilicity. Protein is released when the surface condition becomes superhydrophilic, then resulting in cell detachment [13, 37]. It has been proven previously that the RGD peptide could influence the surface hydrophilicity [38]. However, the result of this study showed that both TN samples and TR samples could have cell detachment with the light power used in the study without a significant difference. This might be explained by the fact that the amount of cells on TN or TR samples was minimal. It has been proven that UV365 illumination is safe for creatures [14], even in a high dose of thousands of mJ/cm^2^ [15]. In this study, it was shown that the number of live cells decreased on both samples when the UV light power was increased to 6.0 mW/cm^2^.

It is known that pseudopodia play an important role in cell functions such as cell adhesion, spreading, and migration [39]. The SEM study showed that the number of pseudopodia was reduced on both samples and cells shrunk and became round after UV365 illumination. This phenomenon was coincident with previous studies [40]. Alongside the changes of cell morphology, the ability of cell adhesion decreased and thus allowed cell detachment to be possible.

The cell sheet reattachment assay, the flow cytometry test of survival of detached cells, and live-dead staining were important indicators of cell sheet activity. Compared to cell sheets from TN samples, cell sheets from TR samples had stronger reattachment ability, less incidence of apoptosis, and fewer dead cells. Previous studies showed that the RGD peptide has the ability to mediate cell apoptosis [41], in which cell adhesion plays an important role. It is known that cell adhesion activates numerous signaling pathways, many of which work on the suppression of apoptosis [42]. In this study, cell sheets from TR samples revealed more living cells and higher activity, indicating that the immobilized RGD peptide used in this study could prevent cell apoptosis, which can increase cell survival and recovery. Additionally, the RGD peptide has been demonstrated to upregulate the expression of certain types of proteins [18]. This prompts that the amount of proteins present on TR samples might be greater than on TN samples. This is coincident with the Western blot result that Collagen I and Runx2 were expressed at a higher level on TR samples than on TN samples. Since proteins have the ability to absorb laser energy [43], cell sheets from TR samples would be illuminated with a lower power of UV365 than TN samples due to the absorption of proteins. This might be another reason that caused a higher cell survival rate on TR samples after UV365 illumination.

In general, the RGD peptide immobilized on TiO_2_ nanodot films can enhance cell adhesion, osteogenic differentiation, and the ability to resist apoptosis on the basis of not affecting the light-induced detachment efficiency.

## 5. Conclusions

This study indicated that immersion was a considerable method to immobilize the RGD peptide on TiO_2_ nanodot films for light-induced detachment technology. Moreover, cell sheets cultured on this kind of biomaterial would be more effective in osteogenesis and resisting cell death. Collectively, this study demonstrated the potential of obtaining cell sheets by light-induced technology as the fundamental element to structure multicoat cell sheets for bone repair.

## Figures and Tables

**Table 1:** Primer list.

Gene	Species	Forward 5′-3′	Reverse 5′-3′
GAPDH	Mouse	ACCCAGAAGACTGTGGATGG	CACATTGGGGGTAGGAACAC
ALP	Mouse	AACCCAGACACAAGCATTCC	GAGAGCGAAGGGTCAGTCAG
Collagen I	Mouse	AGAGCATGACCGATGGATTC	CCTTCTTGAGGTTGCCAGTC
Runx2	Mouse	CAGACCAGCAGCACTCCATA	CAGCGTCAACACCATCATTC
